# Fluoroless ablation of right-sided supraventricular tachycardia: a step-by-step approach and retrospective case series

**DOI:** 10.3389/fcvm.2026.1860451

**Published:** 2026-07-09

**Authors:** David Altmann, Dorian Garin, Etienne Delacrétaz, Stéphane Cook, Mario Togni, Hari Vivekanantham

**Affiliations:** 1 Department of Cardiology, Hospital of Fribourg, Fribourg, Switzerland; 2 Faculty of Science and Medicine, University of Fribourg, Fribourg, Switzerland

**Keywords:** catheter ablation, fluoroless ablation, zero-fluoroscopy, supraventricular tachycardia, electroanatomical mapping, three-dimensional imaging

## Abstract

Fluoroscopy has long been indispensable in electrophysiology procedures. However, the detrimental long-term effects of x-rays on both patients and healthcare workers are well documented. Moreover, the routine wearing of lead aprons is associated with orthopedic complications in the long term. The emergence of new technologies, such as electroanatomic mapping systems and intracardiac echocardiography, has significantly reduced the need for fluoroscopy, thereby alleviating these concerns. This article describes our step-by-step approach to fluoroless catheter ablation of right-sided supraventricular tachycardia and evaluates its implementation in clinical practice through a retrospective case series. Between May 2024 and April 2025, 86 consecutive patients underwent ablation using this strategy. The findings support existing evidence that zero-fluoroscopy interventions are feasible, efficient and safe.

## 1 Introduction

The term supraventricular tachycardia (SVT) comprises a heterogeneous group of arrhythmias involving tissue from the His bundle or above, conventionally excluding atrial fibrillation (1). Paroxysmal SVT, including atrioventricular nodal reentrant tachycardia (AVNRT), atrioventricular reentrant tachycardia (AVRT), atrial tachycardia (AT), has an estimated prevalence of 332.9/100’000 individuals and incidence 57.8/100’000 person-years (2). Atrial flutter (AFL), another common SVT, has an estimated incidence of 88/100’000 person-years, occurs more frequently in males and increases exponentially with age (3). The 2019 European Society of Cardiology guidelines for SVT recommend catheter ablation as first-line treatment for all symptomatic and recurrent reentrant and most focal arrhythmias (1). In Switzerland, the number of catheter ablations has increased almost tenfold over the last 20 years. Despite an observable shift towards more complex procedures in older patients with comorbidities, there has been a decrease in fluoroscopy, ablation and procedure times (4). One of the most important advances in reducing fluoroscopy use has been the introduction of three-dimensional (3D) electroanatomic mapping (EAM) systems, which enable real-time catheter visualization, acquisition of activation and voltage data, and precise navigation within the cardiac chambers without ionizing radiation (5). The well-documented risks associated with radiation exposure, especially the stochastic carcinogenic effects of radiation in both patients and healthcare workers, are the reason for the emergence of the “as low as reasonably achievable” (ALARA) principle. Furthermore, occupational exposure has an impact on both male and female reproductive health, as well as fetal development (6, 7). Apart from eliminating radiation exposure, zero-fluoroscopy also eliminates the need for lead aprons, thereby reducing the risk of associated orthopedic injuries (8). The zero-fluoroscopy approach has been shown in multiple studies to be feasible, efficient and safe (9–13). We have previously described our fluoroless approach guided by intracardiac ultrasound for transseptal access in the setting of pulmonary vein isolation using radiofrequency (RF) (14). In this article, we will focus on right-sided SVTs namely AVNRT, AVRT, AT and AFL.

## 2 Materials and equipment

Our approach is aided by the CARTO™ 3 electroanatomic mapping (EAM) system (Biosense Webster, California, USA). Two to three femoral venous sheaths, ranging in size from 6 Fr to 8 Fr, are inserted depending on the SVT type, as detailed below. A deflectable decapolar catheter (DECANAV® - Biosense Webster, California, USA) and an irrigated ablation catheter (THERMOCOOL SMARTTOUCH® or THERMOCOOL SMARTTOUCH® SF - Biosense Webster, California, USA) are used in all SVT procedures. A quadripolar Josephson-type catheter (Abbott Medical, Plymouth, USA) or a multispline mapping catheter (PENTARAY® or OCTARAY® - Biosense Webster, California, USA) may additionally be used as detailed below.

## 3 Methods

### 3.1 Patient preparation and venous access

The patient is brought to the EP lab in a fasting state. Beta-blockers, calcium channel blockers and anti-arrhythmic drugs are discontinued at least five half-lives prior to the procedure. Local anesthesia is administered in the right groin. Procedural sedation is administered only if necessary and, if required, after completion of the EP study and arrhythmia induction. Right femoral venous access is obtained under ultrasound guidance using Seldinger's technique. A left femoral approach is reserved for cases in which right femoral venous access cannot be obtained. Its use is intended to ensure procedural feasibility rather than to maintain a fluoroless workflow. Heparin is administered at the operator's discretion.

### 3.2 Anatomical mapping and ablation

**Figure 1:**
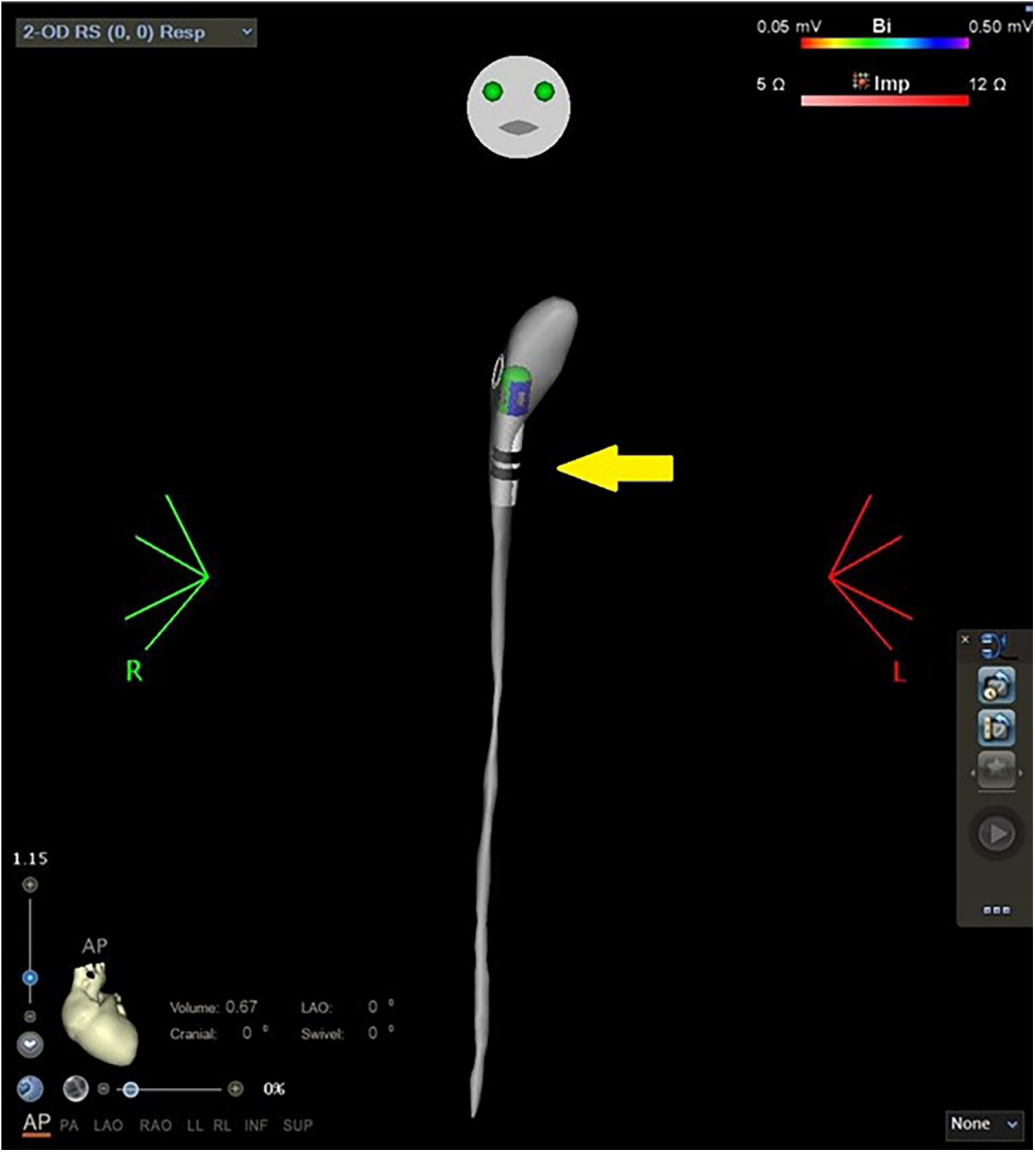
A long sheath is cautiously advanced over the ablation catheter. Reach up to the ablation catheter tip can be confirmed without the use of fluoroscopy by the dark coloring of the proximal electrodes. An antero-posterior cardiac view is shown.

Although a long sheath is not routinely used, a Swartz™ SR0 63 cm 8.5 Fr (Abbott Medical, Plymouth, USA) may be used for slow pathway ablation to improve reach and catheter stability. For other instances, a CARTO VIZIGO™ medium curve steerable sheath (Biosense Webster, California, USA) is used. Approximately half of the long guidewire length is advanced through the right femoral vein without resistance. The long sheath is then advanced over the guidewire, about halfway into the inferior vena cava (IVC), without fluoroscopy. The ablation catheter, which can be visualized on the EAM system, is advanced through the sheath and subsequently “unsheathed” (Figure 1). The entire system can then be cautiously advanced into the right atrium (RA), guided by EAM and appearance of atrial signals.

A THERMOCOOL SMARTTOUCH® SF irrigated catheter is used for mapping and ablation in all cases except for slow pathway ablation, where we prefer using a THERMOCOOL SMARTTOUCH® irrigated catheter. A DECANAV® decapolar mapping catheter is used in all cases and advanced to the heart through the 7 Fr sheath under EAM visualization. Deflection and rotation of the catheter may be required if resistance is encountered, depending on the venous anatomy.

**Figure 2:**
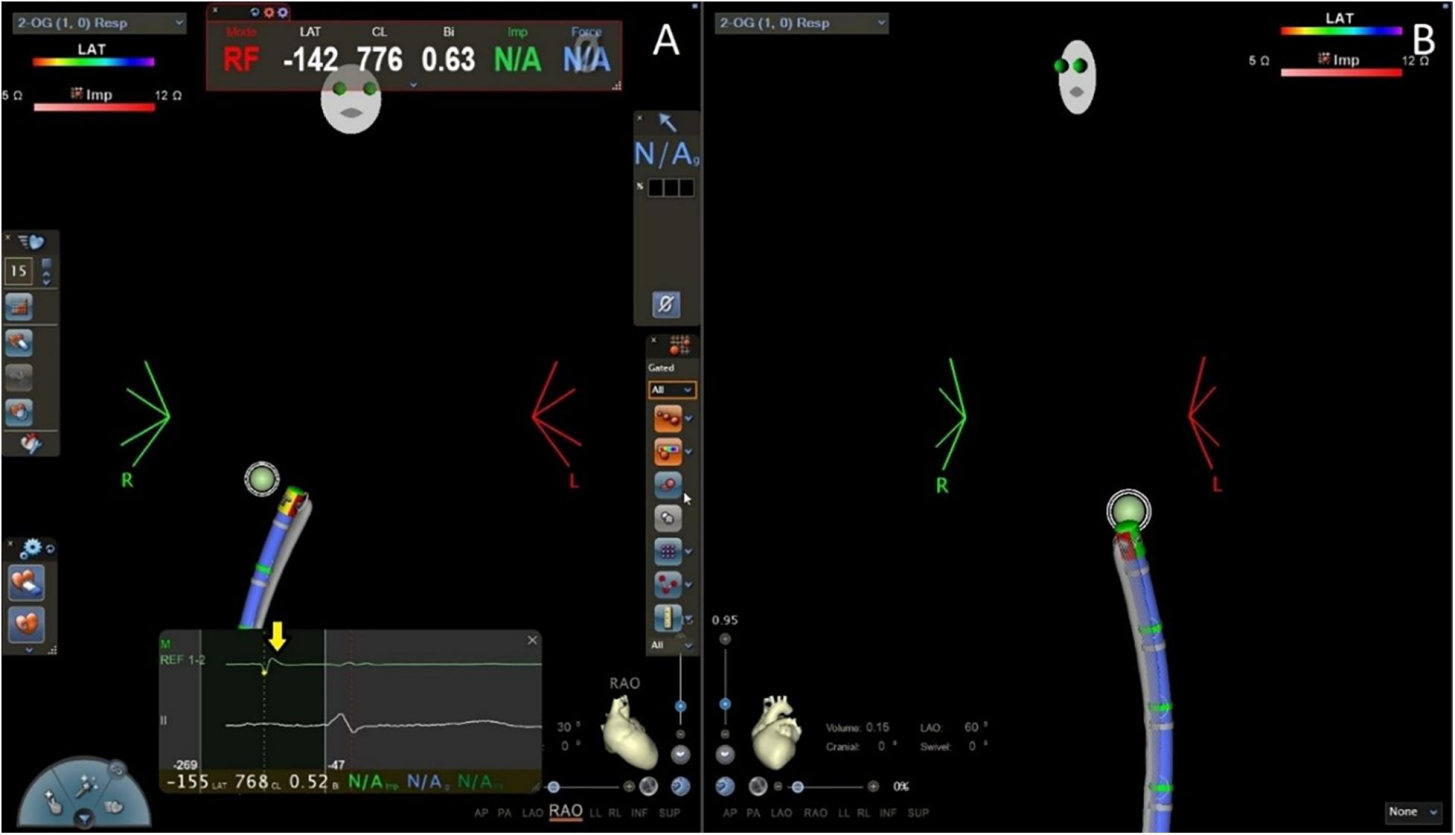
The DECANAV® is advanced, guided by the EAM system, from the right femoral vein up to the inferior vena cava junction (IVC) - right atrial (RA) junction. Presence of an RA electrical signal (yellow arrow) confirms entry into the RA. **(A)** EAM system right anterior oblique (RAO) heart view; **(B)** EAM system left anterior oblique (LAO) heart view.

**Figure 3:**
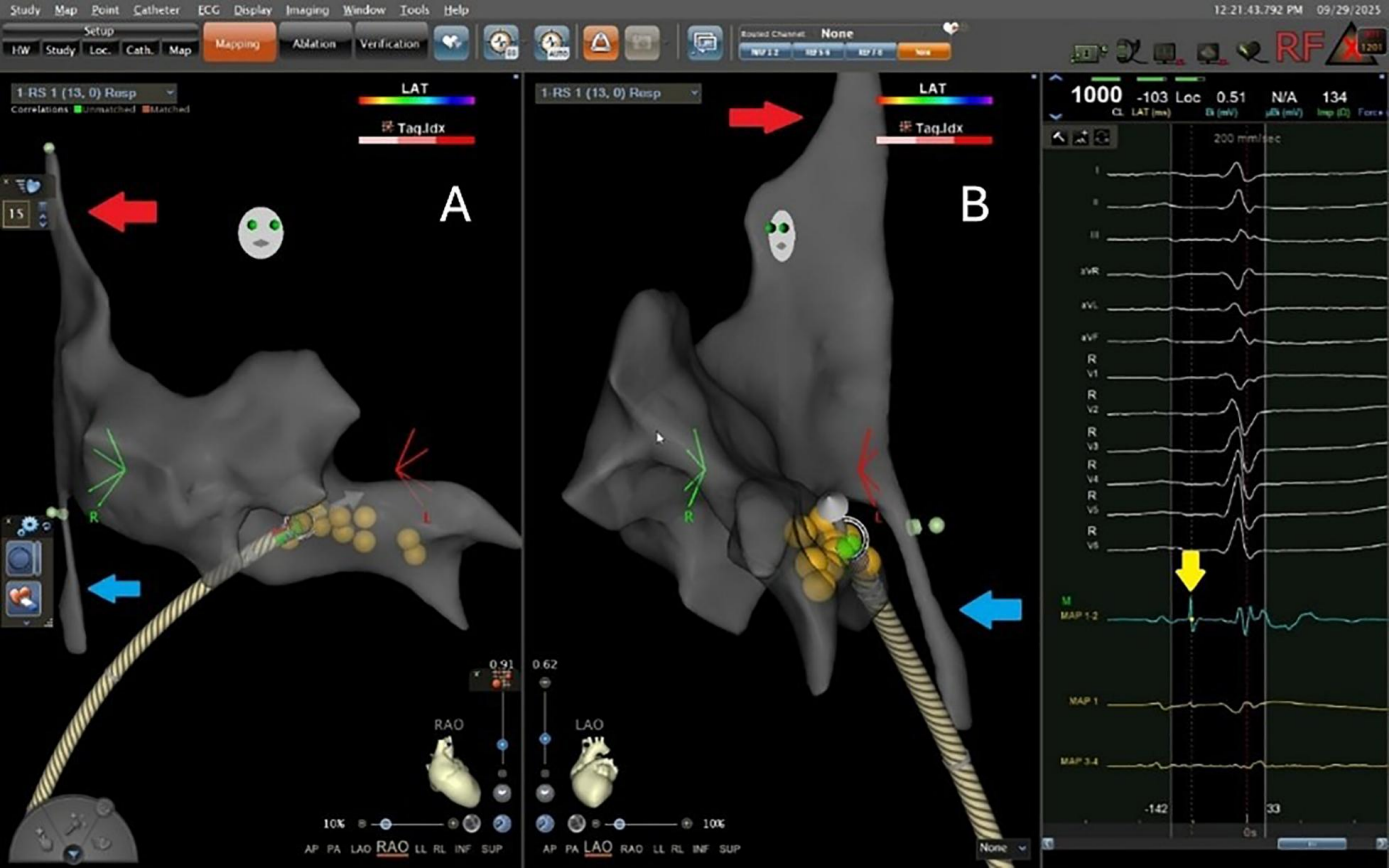
FAM of landmark right-sided cardiac structures such as SVC (red arrow), IVC (blue arrow), His and CS ostium is performed. **(A)** EAM system RAO heart view; **(B)** EAM system LAO heart view.

**Figure 4:**
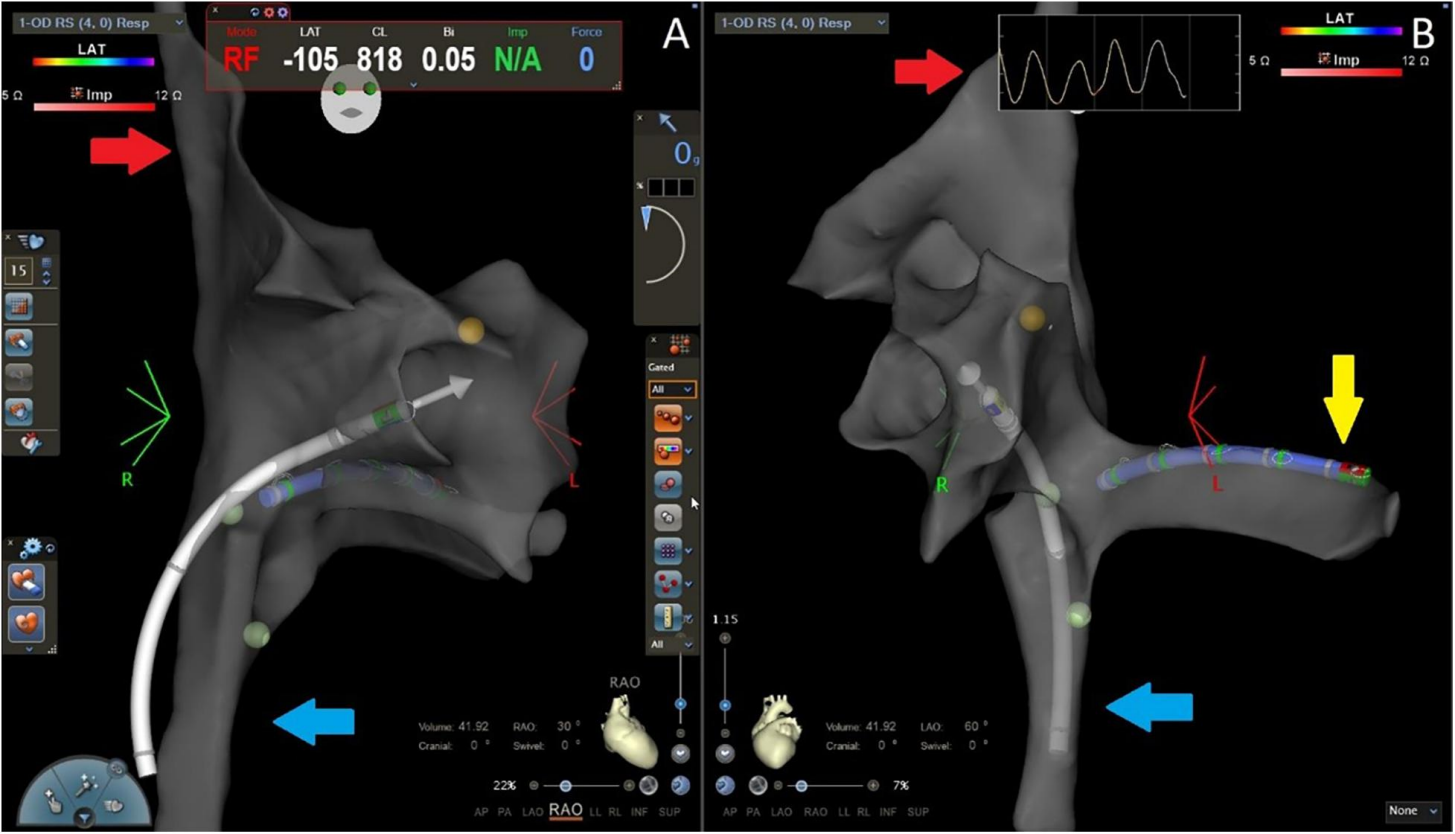
FAM of the RA, IVC (blue arrow), SVC (red arrow) and CS, where a DECANAV® catheter is positioned (yellow arrow). The yellow dot marks the His. **(A)** EAM system RAO heart view; **(B)** EAM system LAO heart view.

Once the IVC-RA junction is reached, the presence of RA electrical signal can be appreciated (Figure 2). Subsequently, Fast Anatomical Mapping (FAM) of the superior vena cava (SVC), RA and coronary sinus (CS) is created using the catheter (Figure 3). The His, which serves as a surrogate marker for the center of the heart, is tagged. The catheter is finally positioned in the CS, where respiratory gating can be performed (Figure 4).

In cases where CS cannulation through the femoral approach is unsuccessful or catheter stability is insufficient, a right jugular venous approach may be used. Following creation of the RA and CS FAM through the jugular approach, fluoroless catheter positioning can be guided by visualization of the catheter bipoles in “ghost mode”.

In cases where the likelihood of diagnosis is low, a quadripolar catheter is used to perform the EP study, before reaching out for an ablation catheter.

#### 3.2.1 Common AFL ablation

**Figure 5:**
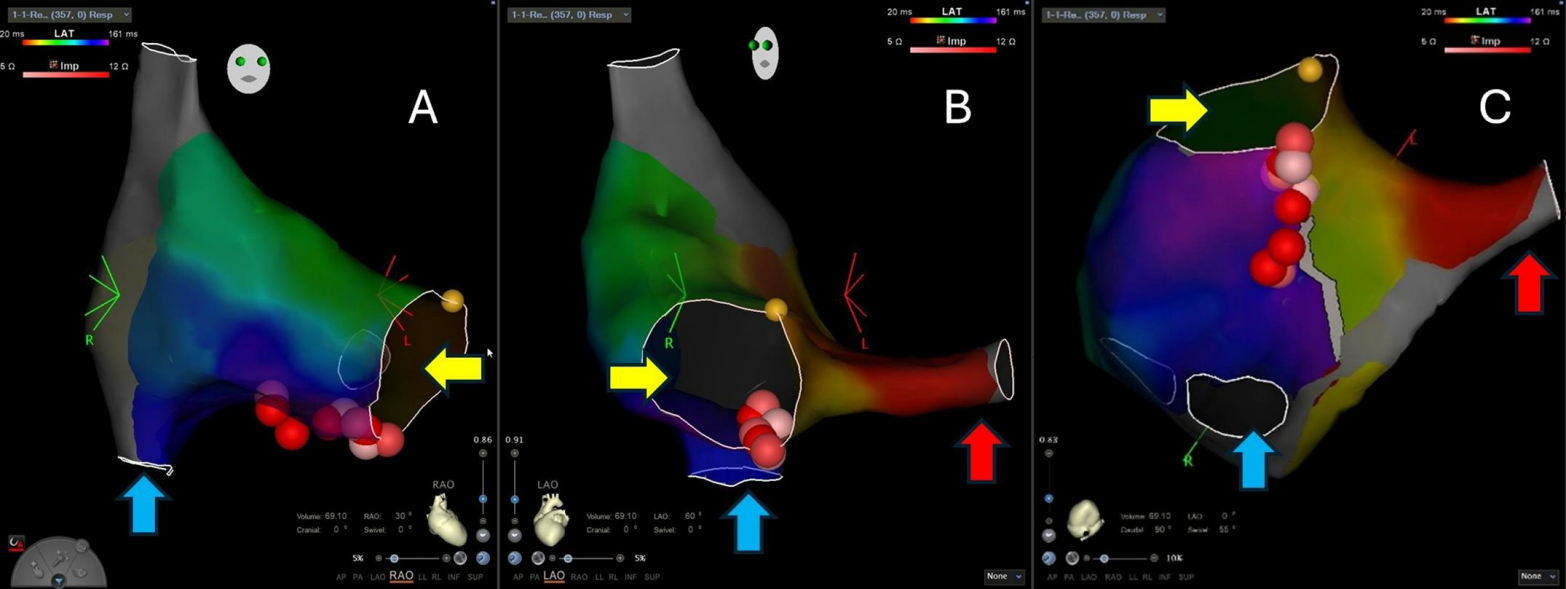
Activation map of the RA during CS ostium pacing, obtained CTI line ablation. The red and pink dots indicate RF applications delivered along the CTI. An earliest (yellow) to latest (purple) atrial activation time rainbow color-coding is used. A counterclockwise RA activation pattern, denoting clockwise atrial activation block at the CTI can be appreciated. Blue arrow: IVC; yellow arrow: right atrioventricular orifice; red arrow: CS. **(A)** EAM system RAO heart view; **(B)** EAM system LAO heart view; **(C)** EAM system inferior LAO view.

The ablation catheter is advanced into the RA through an 8 Fr or a CARTO VIZIGO™ sheath. Following catheter force calibration, FAM of the cavotricuspid isthmus (CTI) is obtained. In the setting of on-going flutter, activation mapping and atrial entrainment are performed from the CTI and CS ostium to confirm typical CTI-dependent flutter. Point-by-point radiofrequency (RF) applications (40W for, 40 s) using 3 mm tags are delivered from the annular to IVC aspect of the CTI. Bidirectional block is confirmed using differential pacing. The map obtained at the end of the procedure is shown (Figure 5).

#### 3.2.2 AVNRT ablation

**Figure 6:**
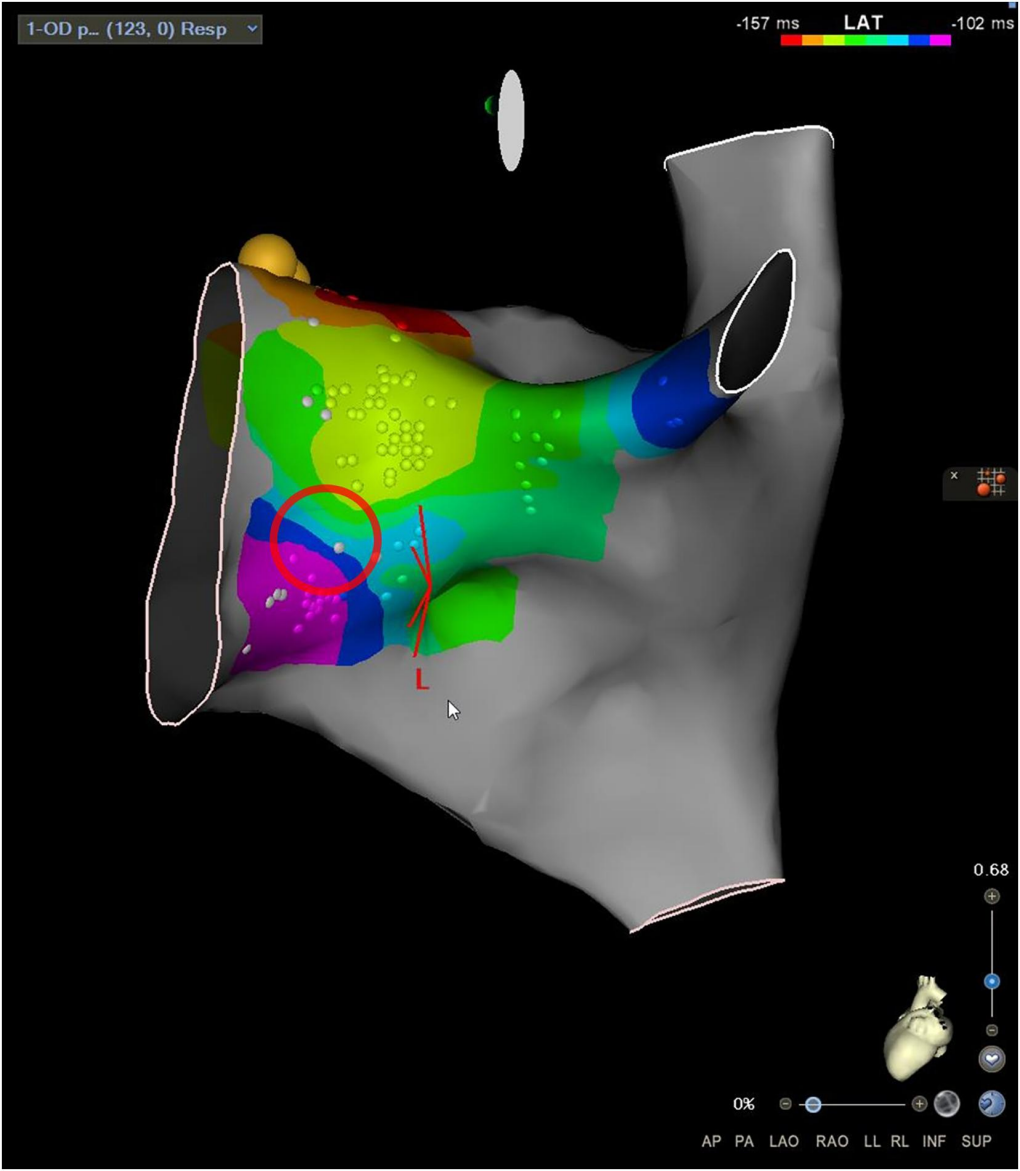
Isochronal activation map of the koch triangle during sinus rhythm. The red circle marks the area of slowest conduction, defined by crowding of 3 isochrones within 1 cm, which is a surrogate marker of the slow pathway.

A two catheter EP study is typically performed. The DECANAV® is positioned in the CS, while the ablation catheter is initially placed at the right ventricular apex (RVA) and subsequently at the His location. An additional quadripolar catheter may be positioned in the RVA depending on the operator's preference. After ruling out the presence of a concealed accessory pathway by RVA pacing, dual atrioventricular (AV) nodal physiology is assessed using decremental atrial pacing and programmed atrial stimulation (PAS). Upon arrhythmia induction, aided by Isuprel and/or atropine administration if required, AVNRT is confirmed using entrainment maneuvers. An isochronal map of the right inferior extension of the slow pathway region is created in sinus rhythm (Figure 6) (15). Ablation is guided by the isochronal map and by the atrial/ventricular signal ratio.

In cases where arrhythmia induction is unsuccessful, empirical slow pathway ablation is performed in the presence of dual AV nodal physiology and tracing highly suggestive of AVNRT.

#### 3.2.3 Focal right AT and macro-reentrant AT ablation

In the absence of ongoing atrial tachyarrhythmia, an atrial burst protocol or programmed atrial stimulation is used for induction, aided by Isuprel infusion if required. An OCTARAY™ multipolar multispline catheter is used for mapping. Its use may be optional in the setting of focal AT, as the ablation catheter alone may suffice for mapping. However, the use of a multipolar catheter is strongly recommended for high-density mapping in cases of atypical or scar-related right-sided AFL. A FAM and activation map of the RA are created. The multipolar mapping catheter is typically exchanged for the ablation catheter, but an additional 8 Fr sheath may be inserted to allow simultaneous catheter use. Subsequently, focal or linear ablation is performed under EAM guidance. Ablation along the RA lateral wall is performed only in the absence of phrenic nerve capture.

#### 3.2.4 Right-sided accessory pathway ablation

An EP study is performed with the DECANAV® catheter positioned in the CS, a quadripolar catheter positioned at the RVA, and the ablation catheter initially placed at the His. The ablation catheter may be switched to the lateral RA during RVA pacing to seek eccentric atrial activation suggestive of an accessory pathway (AP). Mapping and ablation targeting the AP location are performed during atrial and/or RVA pacing, depending on the conduction properties of the pathway (anterograde, retrograde or bidirectional). Alternatively, mapping during AVRT may be performed.

If catheter stability is suboptimal when using the right femoral approach, a right jugular venous approach may provide improved support, particularly for right anterior AP.

### 3.3 Data collection and statistical analysis

Data was collected from the databases of two tertiary cardiology centers between May 2024 and April 2025 (12 months). All ablation procedures were performed by two operators. All right-sided SVT ablation procedures during this period were included, while procedures involving additional ablation targets outside the RA were excluded. Written informed consent was obtained from all participants prior to the procedure. The following procedural parameters were extracted from the ablation protocols: procedure time, radiation exposure, and acute success. Procedure time was defined as the interval from groin puncture to venous sheath removal (skin-to-skin time). Radiation exposure was quantified as dose-area product (DAP) and total fluoroscopy time. Acute success was defined for each type of arrhythmia type in accordance with current international standards.

Data was analyzed using RStudio. Continuous variables are presented as mean ± standard deviation (SD), and categorical variables as n (%). For descriptive comparisons, Student's t-test, Chi-square test or Fisher's exact test were performed as appropriate. Due to small sample sizes, procedural characteristics were reported separately only for the two most common SVT subtypes: AVNRT and CTI-dependent AFL.

## 4 Results

Between May 2024 and April 2025, a total of 96 consecutive patients underwent right-sided SVT ablation procedures. After exclusion of procedures requiring additional ablation targets outside the right atrium and/or a transseptal puncture (3 PVI, 5 left AVRT, 1 left AT, 1 left PVC), 86 patients were included in the final analysis.

### 4.1 Patient population

Baseline characteristics of the study population are presented in Table 1. There were no significant differences in age or sex between the zero-fluoroscopy (ZF) procedures and those requiring conversion to fluoroscopy (Converted). All procedures involving endovascular pacemaker (PM) or implantable cardioverter-defibrillator (ICD) leads were performed with fluoroscopy (*n* = 3). Zero-fluoroscopy was successfully achieved in 71 procedures (81.6%), whereas 16 procedures (18.4%) required conversion to fluoroscopy. The most common indications were AVNRT (*n* = 53, 61%) and CTI ablation (*n* = 29, 33%), with similar distributions between groups. Less frequent indications included atypical AFL (AAFL, *n* = 2), AVRT (*n* = 2), and one electrophysiological study (EPS).

**Table 1:** Population and ablation indications.

Variable	Overall *n* = 86 (100%)	ZF *n* = 71 (82.5%)	Converted *n* = 15 (17.5%)	p-value
Age (years), mean ± SD	59.6 ± 17.5	58.2 ± 17.4	66.1 ± 16.6	0.112
Male sex, *n* (%)	45 (52.3%)	36 (50.7%)	9 (60.0%)	0.711
PM/ICD, *n* (%)	3 (3.5%)	0 (0.0%)	3 (20.0%)	0.009
Indication				0.086
AVNRT	52 (60.5%)	45 (63.4%)	7 (46.7%)	
CTI	29 (33.7%)	23 (32.4%)	6 (40.0%)	
AAFL	2 (2.3%)	2 (2.8%)	0 (0.0%)	
AVRT	2 (2.3%)	0 (0.0%)	2 (13.3%)	
EPS	1 (1.2%)	1 (1.4%)	0 (0.0%)	

ZF, zero-fluoroscopy; Converted, conversion to fluoroscopy; PM/ICD, pace-maker/intracardiac defibrillator; AVNRT, atrioventricular nodal reentrant tachycardia; CTI, cavotricuspid isthmus ablation; AAFL, atypical atrial flutter; AVRT, atrioventricular reentrant tachycardia; EPS, electrophysiological study.

p-value was calculated as appropriate using Welch Two Sample t-test, Pearson's Chi-squared test or Fisher's exact test.

### 4.2 Procedure characteristics

Reasons for conversion to fluoroscopy are summarized in Table 2. In two cases, brief fluoroscopic acquisition was used to allow integration of the EAM heart view with fluoroscopic imaging using the CARTOUNIVU™ module (Biosense Webster, California, USA). Procedural characteristics are presented in Table 3. Acute procedural success was achieved in all but one case (98.8%). Procedures requiring conversion to fluoroscopy were not associated with longer procedure times compared with zero-fluoroscopy cases. Differences between the two main indications, AVNRT and CTI-dependent AFL, are shown in Table 4. No immediate complications were reported for all procedures.

**Table 2:** Reasons for conversion to fluoroscopy.

Reason	n (%)
PM/ICD leads	3 (20.0)
CS positioning/cannulation	3 (20.0)
Iliac vein tortuosity	2 (13.3)
Difficult anatomy	2 (13.3)
CARTOUNIVU™ guidance	2 (13.3)
Catheter positioning in right ventricle	1 (6.7)
Difficulty achieving CTI block	1 (6.7)

CS, coronary sinus; others, see Table 1.

**Table 3:** – procedure characteristics.

Characteristic	Overall (*n* = 86)	ZF (*n* = 71)	Converted (*n* = 15)	p-value
Procedure time (min)	60.9 ± 21.9	58.9 ± 20.0	70.1 ± 28.3	0.166
Fluoroscopy time (s)			124.2 ± 104.1	
Radiation dose DAP (cGy·cm^2^)			109.9 ± 111.7	
Acute success^a^	84 (98.8%)	69 (98.6%)	15 (100.0%)	

DAP, dose area product; others, see Table 1.

a EPS were excluded for this calculation.

**Table 4:** procedure characteristics of the main indications: AVNRT and CTI ablation.

Indication	Group	Procedure time (min)	Fluoro time (s)	DAP (cGy·cm^2^)
AVNRT	All (*n* = 52)	59.1 ± 18.0	13.4 ± 37.3	6.0 ± 20.1
	ZF (*n* = 45)	60.0 ± 18.4		
	Converted (*n* = 7, 13%)	53.6 ± 15.3	62.7 ± 61.7	44.7 ± 37.6
		*p* = 0.343		
CTI	All (*n* = 29)	58.1 ± 23.3	42.2 ± 94.7	39.4 ± 98.0
	ZF (*n* = 23)	53.8 ± 20.9		
	Converted (*n* = 6, 21%)	74.3 ± 26.8	185.8 ± 117.0	190.6 ± 138.8
		*p* = 0.127		

p-value was calculated using the Welch Two Sample t-test.

see Tables 1, 3.

## 5 Discussion

Our findings demonstrate a low conversion rate to fluoroscopy, with no apparent disadvantages in terms of procedural duration or acute success, and without safety concerns compared with conventional fluoroscopy-guided approaches. This strategy allows the consistent and reproducible implementation of zero-fluoroscopy procedures in routine clinical practice. These results are consistent with a growing body of evidence demonstrating the feasibility, efficacy, and safety of fluoroless ablation procedures (9–11). Focusing on SVT ablation, important studies have described fluoroless ablations of AVRT and AVNRT using the Ensite NavX (St. Jude Medical, St. Paul, MN, USA) system for navigation (12, 16). Hofer et al. reported outcomes most comparable to the present study by demonstrating the feasibility, efficiency and safety of right-sided arrhythmias using only CARTO 3 for navigation (13).

Despite the overall high feasibility of this fluoroless approach, the use of fluoroscopy remains necessary in selected cases. In patients with PM/ICD, a fluoroscopy-guided approach is preferred from the beginning in order to minimize the risk of lead dislodgement. For the other cases, the most common reasons for conversion were anatomical challenges, including difficulties with CS cannulation and positioning, iliac vein tortuosity or anatomy perceived as unusual by the operator. The likelihood of conversion in these situations is strongly influenced by operator experience and familiarity with fluoroless techniques. In this context, Pani et al. demonstrated that fluoroscopy time for SVT ablations decreases significantly with increasing operator experience and trust in the zero-fluoroscopy technique (17).

To achieve zero-fluoroscopy, several technological tools are required. The development of 3D EAM systems has been fundamental for minimal- and zero-fluoroscopy strategies (5). Information provided by contact force-sensing catheters is crucial for safely reducing fluoroscopy time, though non-contact force catheter are available for the same purpose (18).

For more complex procedures, such as those requiring transseptal puncture or a retrograde aortic approach, intracardiac echocardiography (ICE) may be necessary to safely achieve zero-fluoroscopy. Such scenarios are rarely encountered in right-sided procedures. Additionally, routine ICE use is associated with increased costs and requires an additional venous access and indwelling catheter, potentially increasing the risk of complications (13). For these reasons, our strategy relies primarily on EAM, with conversion to fluoroscopy when necessary.

A fluoroscopy-guided approach is preferred in patients with PM/ICD to avoid lead dislodgement. Shimamoto et al. demonstrated that zero-fluoroscopy is feasible in these settings when ICE is combined with careful catheter manipulation (19). Such procedures require substantial operator experience with both ICE-guided navigation and lead management, and evidence regarding long-term outcomes and safety remains limited. For these reasons, fluoroscopy was used in all patients with PM/ICD included in this study.

Beyond minimizing the occupational hazard of radiation exposure, the introduction of apron-free procedures has had a positive impact on working conditions of all members of the EP lab.

### 5.1 Limitations

It is important to note that no selection process was applied. The described workflow represents our standard approach and was consistently used in all procedures, both during and beyond the study period. As such, our data reflects an unselected, real-world patient population. Consequently, no control group undergoing a conventional or minimal fluoroscopy approach was included. Missing data could not be retrieved or analyzed. The overall number of cases was modest. Safety assessment was limited to immediate periprocedural complications occurring within the first hours after the procedure. While no major complications were reported based on the operator's knowledge and feedback received from patients and/or treating physicians, the absence of formal mid- or long-term clinical follow-up remains an important limitation of our data. Limited follow-up remains a common limitation in the current literature on fluoroless ablation procedures. Overall, our data are primarily descriptive and serve to support existing evidence.

### 5.2 Outlook

When considering the development of fluoroless procedures, it appears that, provided sufficient resources and expertise are available, the technical limitations of zero-fluoroscopy are minimal, particularly for RA ablation. Nevertheless, there is substantial heterogeneity in fluoroscopy use, even within healthcare systems, where access to advanced mapping technologies is widely available. Further research is needed to better understand the factors influencing fluoroscopy use and facilitate broader and more consistent implementation of fluoroless strategies.

Transitioning to fluoroless procedures represents a gradual process that depends on habit and structured training. Orientation and troubleshooting techniques require experience with non-fluoroscopic navigation. Although zero-fluoroscopy is feasible in the majority of cases, the immediate availability of fluoroscopy as a backup remains essential in the event of procedural challenges or complications.

However, even this paradigm may evolve in the future. A recent Chinese expert consensus has described the potential for constructing fluoroless EP laboratories under specific conditions (20). While the availability of mobile digital subtraction angiography within the laboratory is still recommended, a completely fluoroscopy-free environment may be possible, provided that a rapid and safe transfer to fluoroscopic imaging can be ensured if needed.

## Conclusion

Reducing radiation exposure in healthcare is essential to ensure long-term safety for both patient and medical staff. Fluoroless catheter ablation of right-sided SVT is feasible, safe and effective in routine clinical practice when supported by appropriate technology and operator experience.

## Data Availability

The raw data supporting the conclusions of this article will be made available by the authors, without undue reservation.
